# Polymorphisms of *RAD51B* are associated with rheumatoid arthritis and erosion in rheumatoid arthritis patients

**DOI:** 10.1038/srep45876

**Published:** 2017-03-31

**Authors:** Liqiang Zhi, Shuxin Yao, Wenlong Ma, Weijie Zhang, Honggan Chen, Meng Li, Jianbing Ma

**Affiliations:** 1 Department of Joint Surgery, Honghui Hospital, Xi’an Jiaotong University Health Science Center, Xi’an, Shaanxi, China; 2 Department of Hip Injury and Disease, Orthopedic Hospital of Henan Province, Qiming South Road No.82, Luoyang, Henan, China; 3 Department of Orthopedics, the First Affiliated Hospital, Xi’an Jiaotong University, Xi’an, Shaanxi, China

## Abstract

Rheumatoid arthritis (RA) is a common, chronic autoimmune disease affecting 0.5–1.0% of adults worldwide, including approximately 4.5–5.0 million patients in China. The genetic etiology and pathogenesis of RA have not yet been fully elucidated. Recently, one new RA susceptibility gene (*RAD51B*) has been identified in Korean and European populations. In this study, we designed a two-stage case-control study to further assess the relationship of common variants in the *RAD51B* gene with increased risk of RA in a total of 965 RA patients and 2,511 unrelated healthy controls of Han Chinese ancestry. We successfully identified a common variant, rs911263, as being significantly associated with the disease status of RA (*P* = 4.8 × 10^−5^, OR = 0.64). In addition, this SNP was shown to be related to erosion, a clinical assessment of disease severity in RA (*P* = 2.89 × 10^−5^, OR = 0.52). These findings shed light on the role of *RAD51B* in the onset and severity of RA. More research in the future is needed to clarify the underlying functional link between rs911263 and the disease.

Rheumatoid arthritis (RA) is a common, chronic autoimmune disease characterized by irreversible joint damage and deformity that affects 0.5–1.0% of adults worldwide, including approximately 4.5–5.0 million patients in China^1^,^2^,^3^,^4^,^5^,^6^,^7^. In addition to synovial inflammation and hyperplasia and destruction of cartilage and bone, the presence of serum autoantibodies, rheumatoid factor (RF) and anti-cyclic citrullinated peptides (anti-CCP) is characteristics of RA^8^,^9^. The progressive joint disability in RA severely impact the quality of life and socioeconomic status of patients and their families, constituting a major public health issue. Although the mechanisms underlying the pathogenesis of RA have not yet been fully elucidated, many studies have demonstrated that RA is a multifactorial disorder caused by complex interactions of multiple susceptibility genes, environmental factors, and exposure to certain infections^10^,^11^,^12^. Over the past decades, substantial information has been generated on genetic factors contributing to the elusive etiology of RA, with a heritability of approximately 65%^12^. Genetic association analyses that include genome-wide association studies (GWASs) have reported reliable data related to genetic susceptibility of RA, and previous large-scale investigations have also identified that RA shares common genetic causative factors in different ethnic population^13^,^14^. To date, more than 100 susceptibility loci are associated with RA^15^. Although GWAS has provided a powerful approach for genetic studies of complex diseases^16^,^17^,^18^,^19^,^20^,^21^, the results account for only a small percentage of the estimated heritability, with few systematic biological interpretations^22^. Nonetheless, it is known that significant differences exist with regard to the contribution of common variations in susceptibility genes among different ethnic populations; therefore, follow-up studies are essential to confirm previous findings and extend them to different populations.

Recently, a new single-nucleotide polymorphism (SNP) (rs911263) within the *RAD51B* gene was identified as being associated with increased RA risk in Koreans and Europeans^23^,^24^. The *RAD51B* gene, located at 14q24.1, encodes a member of the RAD51 protein family. *RAD51B*, a known RAD51 paralog, exhibits central recombinase activity in mammalian cells^25^,^26^. It was also reported that *RAD51B* plays an important role in homologous recombinational repair (HRR) of DNA double-strand breaks (DBSs) to maintain cell genomic stability and is a promising candidate oncogene and biomarker for cancer diagnosis and prognosis^24^,^27^,^28^,^29^. Indeed, the absence of *RAD51B* may disrupt the formation of *RAD51B* nucleoprotein filaments, the initial stage of HRR, thereby resulting in DNA mutations, rearrangements and/or loss of chromosomes^27^. *RAD51B* has been shown to form a stable heterodimer with the family member RAD51C, which further interacts with other family members such as RAD51, XRCC2, and XRCC3. Because the underlying biological mechanisms of RA remain largely unknown, the effects of *RAD51B* on RA have not been clarified, despite the evidence of strong significant associations within Korean and European populations^8^,^13^,^14^,^30^.

Considering that the role of *RAD51B* in RA susceptibility in Han Chinese has not been assessed, we performed a two-stage case-control study to evaluate the transferability of discovered RA susceptibility loci in Han Chinese individuals to improve our current understanding of the role of the *RAD51B* gene in predisposition to RA. Moreover, there are no reports on the association between *RAD51B* and clinical manifestations of RA, such as the 28-joint disease activity score (DAS28) and clinical severity. The other aim of our study was to assess the role of potential associated variants in the clinical manifestations of RA, which may help in defining the primary set of risk alleles for RA susceptibility and provide clues to the mechanisms involved in the etiology and pathogenesis of RA.

## Materials and Methods

### Subjects

Two independent cohorts of RA patients and controls were included in this study. In the discovery stage, we recruited 402 RA patients (age 33–61 years) and 969 unrelated healthy controls (age 33–61 years) from Honghui Hospital and the First Affiliated Hospital of Xi’an Jiaotong University. In the replication stage, 2,105 subjects consisted of 563 RA patients (age 36–64 years) and 1,542 unrelated healthy controls (age 36–64 years) who were enrolled from Orthopedic Hospital of Henan Province. All subjects included in the study were random chosen genetically unrelated Han Chinese individuals without migration history within the previous three generations. All patients were diagnosed with RA according to the 2008 Classification Criteria of the American College of Rheumatology, and all healthy controls had no history of rheumatism or infectious or chronic inflammatory autoimmune diseases. This study was performed in accordance with the ethical guidelines of the Helsinki Declaration of 1975 (revised in 2008) and was approved through the Local Ethics Committee of Xi’an Honghui Hospital. Informed consent was obtained from subjects.

### Clinical assessments

The history of all RA patients was recorded, especially regarding presenting symptoms, joints affected and extra-articular features, and medications. In assessing disease activity according to the DAS28, it was determined that RA patients had been not treated with intra-articular corticosteroids, MTX or biological agents. Joint erosion in RA patients was evaluated by X-rays of the hands and feet. We obtained only the presence or absence of erosion, without radiological score calculation. Patients were assigned to two groups according to erosion (erosive RA and non-erosive RA). Moreover, they were grouped according to the presence or history of extra-articular features. Laboratory parameters were recorded, including rheumatoid factor (RF), anti-cyclic citrullinated peptide antibody (aCCP), anti-glucose phosphate isomerase (aGPI) and the erythrocyte sedimentation rate (ESR). In addition, we also obtained the information of visual analogue scale (VAS) from each RA patients. Demographic information was obtained from each subject at enrollment.

### SNP selection and genotyping

As an initial screen of common SNPs in the Han Chinese population, we searched for all SNPs with a minor allele frequency (MAF) ≥ 0.05 of the *RAD51B* gene in the 1000-genomes CHB database. Then, MAF ≥ 0.05 with pair-wise tagging and r^2^ ≥ 0.5 were used as cutoff criteria during tag SNP selection, resulting in 62 tag SNPs covering the *RAD51B* region (Supplementary Table S1).

Peripheral venous blood samples were collected in plain tubes, and genomic DNA was isolated from peripheral blood leukocytes according to the manufacturer’s protocol (Genomic DNA kit, Axygen Scientific, Inc., CA, USA). SNP genotyping was performed using the high-throughput Sequenom MassARRAY platform with iPLEX GOLD chemistry (Sequenom, San Diego, CA, USA) based on the manufacturer’s protocols^31^. The results were processed using Sequenom Typer 4.0 software, and genotype data were generated from the samples^32^. The status of the case and control samples was blinded for quality control during genotyping processes, and random processing of 5% of samples was performed with a concordance of 100%.

### Statistical analyses and power analyses

#### Power Analyses

To estimate the statistical power of our study design, we implemented a comprehensive power analysis using Genetic Power Calculator^33^. The results of this power analysis are summarized in Supplemental Table S2. As shown, if the underlying risk allele of RA has an MAF of ~0.1 and OR greater than 1.3, our sample will achieve statistical power of >0.8.

#### Genetic Association Analyses

We conducted association analyses at two levels: the single-marker level and the haplotype level. For single-marker-level analyses, we conducted logistic regression for each SNP marker to evaluate their underlying effects on the onset of RA. The SNP markers were coded in three modes: additive, dominant and recessive. In each logistic model, sex and age were included as two covariates to remove potential confounding effects. We implemented a two-stage study design. In the discovery stage, we tested all 62 tag SNPs. In the validation stage, we only included those SNPs with nominal significance in the discovery stage (and SNPs strongly related with these SNPs). Bonferroni corrections were applied to genetic association analyses. The *P* value threshold in the discovery stage was 0.0008 (0.05/62). For haplotype-level analyses, linkage disequilibrium (LD) blocks were constructed for the 62 SNPs selected for the discovery stage. Because analyses of several SNPs are insufficient to draw a conclusion^34^,^35^,^36^, haplotype-based analyses were then conducted according to these LD blocks. In addition, we also performed haplotype-based analyses for all the SNPs selected for genotyping in validation stage.

In addition to genetic association analyses targeting the disease status of RA, we also conducted association analyses between significant SNPs (with RA status) and three RA-related indicators or phenotypes: VAS, DAS28, erosions and extra-articular involvement. DAS28 is an important clinical indicator measuring the disease activity of RA. A DAS28 score greater than 5.1 is considered to be indicative of high disease activity, between 5.1 and 3.2 of moderate disease activity and less than 3.2 of low disease activity Erosions and extra-articular manifestations are two clinical assessments of the severity of RA. RA with erosions and extra-articular manifestations were considered to be the severe type. Only RA patients (965) were included in these analyses. For quantitative traits (DAS28), linear regression was implemented; for qualitative traits such as erosions and extra-articular manifestations, logistic models were fitted. Age and sex were also included as covariates in the model fitting. All these genetic association analyses were implemented by Plink^37^. The regional association plot was generated using LocusZoom^38^.

#### Bioinformatic Analyses and Data Mining

The web-based population genetics software SNAP^39^ was utilized to identify SNPs that were not genotyped in this study but in strong LD in the Chinese population with significant SNPs. Data from the Chinese population in the 1000 genomes project were used as the reference in this analysis. To predict the potential functional significance of SNPs (especially for intronic/synonymous SNPs), we utilized RegulomeDB, a database that annotates SNPs with known and predicted regulatory elements in intergenic regions of the *Homo sapiens* genome^40^. In addition, STRING^41^, a functional protein-protein interaction network database, was utilized to investigate the network neighbors of our candidate gene *RAD51B*.

## Results

### Characteristics of the subjects

A total of 965 RA patients and 2,511 healthy controls were included in our two-stage case-control study. In each stage, the RA patients and healthy controls were matched by mean age, and there were no significant differences in gender distribution between the cases and controls (Table 1). The demographic and clinical data of the RA patients are presented in Table 1.

### Genetic association analyses with RA status

**Figure 1:**
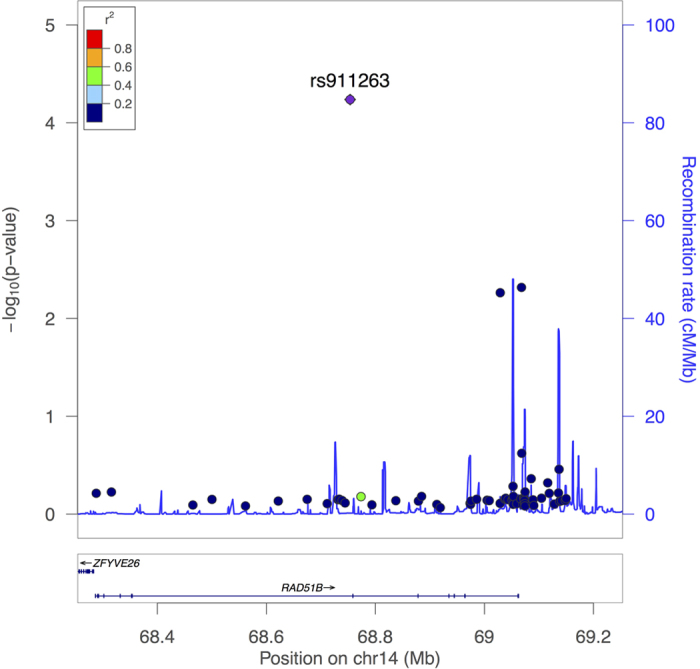
Regional association plot based on the association study results of 62 SNPs in discovery stage.

Three SNPs (rs911263, rs2525504, rs17756404) were identified to be nominally significant in the discovery stage. These SNPs and 4 other SNPs that are strongly correlated with them were genotyped and analyzed in the validation stage (Table 2), and only one, rs911263, was successfully validated (*P* = 4.8 × 10^−5^). The C allele of this SNP showed a strong protective effect on RA (OR = 0.64). The association results of the discovery stage based on 62 SNPs are shown in Fig. 1. As shown in this regional association plot, most of the SNPs were not related to rs911263. The complete results of single-marker-based analyses in the discovery stage are summarized in Supplemental Table S3. Two 2-SNP LD blocks were constructed for haplotype analyses based on the discovery dataset, but they were not associated with RA status in our sample (Supplemental Table S4). In addition, haplotype based analyses using combined data for all 7 SNPs were summarized in Supplemental Table S5, which indicated a similar association pattern with single marker based analysis.

### Genetic association analyses with disease severity and activity indicators

We implemented association analyses between the significant SNP rs911263 and four disease activity- and severity-related clinical assessments of RA. Our results indicated that one, erosion, was significantly associated with this SNP (*P* = 2.89 × 10^−5^). Our finding showed that the C allele of rs911263 is associated with a lower incidence rate of erosion in RA patients (OR = 0.52). The complete results of these association analyses can be found in Table 3.

### Bioinformatic analyses

Using SNAP and 1000 genomes project data, we identified 3 ungenotyped SNPs, rs3784099, rs7148416 and rs10129646, as being in strong LD in the Asian population with our significant SNP rs911263. None of these 3 SNPs or rs911263 are exonic. Their potential functional significance was evaluated using RegulomeDB, which has a systematic score system, whereby an SNP is assigned a score ranging from 1 to 6: the lower the score is, the more functional significance the SNP might have. A score of 4 was found for our targeted SNP rs911263, and the other three SNPs, rs3784099, rs7148416 and rs10129646, had scores of 3, 6 and 4, respectively. All four SNPs showed moderate functional significance.

**Figure 2:**
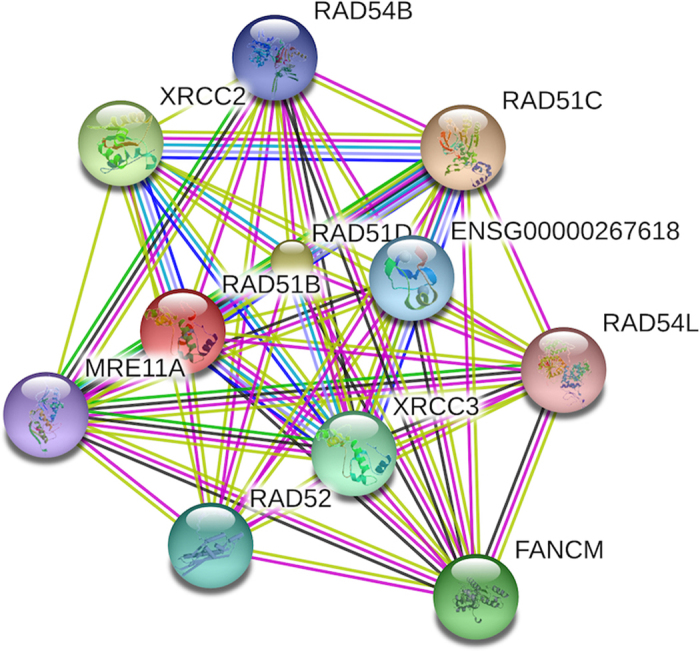
Protein-protein interaction (PPI) patterns of *RAD51B*. A set of 10 genes, including the RAD gene family (*RAD51D, RAD51C, RAD52, RAD54 L* and *RAD54B*), have strong evidence of PPI with *RAD51B*.

We also investigated protein-protein interaction network neighbors of our candidate gene *RAD51B* and identified 10 other genes with strong interactions with *RAD51B*. Among them, *RAD51D, RAD51C, RAD52, RAD54 L* and *RAD54B*, belong to the RAD gene family (Fig. 2).

## Discussion

Multiple previous studies have indicated a connection between *RAD51B* and RA. In a meta-analysis conducted by McAllister *et al*.^23^, rs911263 in *RAD51B* was identified as being significantly associated with RA susceptibility. The protective effect of the C allele (or the G allele if using a different reference) in that study was identified as approximately 0.8; however, our finding indicated that this effect can be as high as 0.5–0.6. The difference between our study and that of McAllister *et al*. can be explained by the difference in genetic background of the study subjects. The meta-analysis was based on a sample of Europeans, whereas our study was based on the Chinese Han population. In addition, a study based on Korean and European populations also identified a significant association between rs911263 and RA susceptibility^30^. Compared to these previous studies, one advantage of our study is that we did not only check the association between rs911263 and RA status but investigated the potential link between this SNP and disease activity and RA severity in patients. Our findings regarding the connection between rs911263 and erosions indicated that the C allele of rs911263 is associated with a lower incidence rate of erosion in RA patients. To our knowledge, this finding has not been reported before. Further replication of our results in other populations is needed.

*RAD51B* is an important member of the RAD51 protein family, which are evolutionarily conserved proteins essential for DNA repair via homologous recombination. The function of this gene is rather fundamental in human metabolism, which may partly explain why the *RAD51* family is evolutionarily conserved: any mutations with high functional significance might be lethal. The SNP rs911263 has been identified as significantly associated with RA susceptibility in multiple previous studies, and this was validated in our large sample based on the Han Chinese population. Therefore, the chance that this is merely a false positive signal duet to confounding factors is very low. The next question to address is how this SNP affects RA susceptibility. Three hypotheses can be invoked. The first is that rs911263 has direct functional significance and thus could have a direct effect on the transcription or translation of *RAD51B*. However, our investigation using RegulomeDB for this SNP does not support this: rs911263 showed only moderate functional significance with a score of 4. Another hypothesis is that rs911263 is simply a surrogate for some underlying common SNPs not genotyped in our study. Again, scrutinizing the potential functional significance of the three other common SNPs in strong LD with rs911263 (rs3784099, rs7148416 and rs10129646) tends to negate this hypothesis. Our findings showed that, similar to rs911263, these three common SNPs had only moderate functional significance (RegulomeDB scores ranging from 3 to 6). The last hypothesis is that SNP rs911263 is a surrogate for a combination of multiple rare variants. However, due to the limitation of our study, it is difficult to validate this hypothesis. More studies, especially those employing sequencing technology, which can provide information for both rare and common variants, should be conducted to examine the direct link between the association signal and functional effects of this SNP on RA onset.

Despite the advantages of our study described above, there are also several limitations. First, population stratification is one of the most important confounding factors for most population-based genetics studies. In GWASs, this confounding factor can be adjusted by principle component analysis (PCA), which requires thousands and even tens of thousands of markers. Due funding limitations, it was impossible for us to conduct PCA to adjust population stratification. Instead, we implemented certain criteria to confine the genetic background of our study subjects during the sample recruitment stage to avoid the potential population stratification^42^,^43^. Another limitation is that we only evaluated one gene: *RAD51B*. However, human metabolism and disease onset are complex processes that might involve multiple related genes and several functionally related pathways^44^. Thus, it may be necessary for researchers to thoroughly investigate the entire RAD51 gene family and *RAD51B* network neighbor genes in the future.

In summary, we investigated the potential association between common polymorphisms in *RAD51B* and RA susceptibility in the Chinese Han population. We successfully identified an intronic SNP, rs911263, as being significantly associated with the disease status of RA in our study subjects. Furthermore, we investigated the potential connection between this SNP and certain disease activity and severity indicators of RA. Our results indicated that SNP rs911263 is significantly associated with erosions occurring in RA patients. Despite these statistical findings, more research in the future is needed to clarify the underlying functional link between rs911263 and RA.

## Additional Information

**How to cite this article**: Zhi, L. *et al*. Polymorphisms of *RAD51B* are associated with rheumatoid arthritis and erosion in rheumatoid arthritis patients. *Sci. Rep.* **7**, 45876; doi: 10.1038/srep45876 (2017).

**Publisher's note:** Springer Nature remains neutral with regard to jurisdictional claims in published maps and institutional affiliations.

## Supplementary Material

Supplementary Information

## Figures and Tables

**Table 1:** The Characteristics of RA patients and healthy controls.

Characteristics	Patients (N = 402)	Controls (N = 969)	*P*-value (χ^2^ or t)	Patients (N = 563)	Controls (N = 1,542)	*P*-value (χ^2^ or t)
	Discovery stage			Replication stage		
Age (years)	50.46 ± 7.74	50.18 ± 8.07	0.551 (−0.597)	52.37 ± 8.33	52.16 ± 8.25	0.604 (−0.519)
Gender (female/male) (%)	315/87 (78.4%/21.6%)	756/213 (78.0%/22.0%)	0.890 (0.019)	416/147 (73.9%/26.1%)	1137/405 (73.7%/26.3%)	0.943 (0.005)
Disease duration (months)	48.37 ± 20.42	NA	NA	50.23 ± 24.38	NA	NA
RF seropositivity [n (%)]	343 (85.32%)	NA	NA	468 (83.13%)	NA	NA
Anti-CCP positive [n (%)]	312 (77.61%)	NA	NA	441 (78.33%)	NA	NA
Anti-GPI positive [n (%)]	249 (61.94%)	NA	NA	352 (62.52%)	NA	NA
ESR (mm/h)	41.27 ± 18.42	NA	NA	43.79 ± 18.58	NA	NA
VAS	44.19 ± 3.43	NA	NA	42.90 ± 3.07	NA	NA
DAS28 score	6.16 ± 0.60	NA	NA	5.94 ± 0.54	NA	NA
Patients with erosions [n (%)]	238 (59.20%)	NA	NA	312 (55.42%)	NA	NA
Patients with extra-articular features [n (%)]	52 (12.94%)	NA	NA	55 (9.77%)	NA	NA

Data were shown as mean ± SD, except gender. NA, not applicable; RA, rheumatoid arthritis; RF, rheumatoid factor; anti-CCP, anti-cyclic citrullinated peptide antibody; anti-GPI, anti-Glucose phosphate isomerase; ESR, erythrocyte sedimentation rate; VAS, visual analogue scale; DAS28, 28-joints disease activity score.

**Table 2:** Summarized results of the association analyses on RA status for SNPs included in the validation stage.

CHR	SNP	BP	A1	Discovery Stage			Validation Stage		
				OR	STAT	*P*	OR	STAT	*P*
**14**	**rs911263**	**68286876**	**C**	**0.58**	**−4.02**	**5.79 × 10**^**−5**^	**0.64**	**−4.07**	**4.80 × 10**^**−5**^
14	rs911256	68306863	C	0.93	−0.44	0.6635	0.91	−0.72	0.4709
14	rs17105837	68562024	A	0.98	−0.28	0.7774	0.98	−0.22	0.8261
14	rs2525504	68562256	G	0.78	−2.78	0.0055	0.87	−1.94	0.0521
14	rs4531674	68600026	A	1.05	0.38	0.7016	0.96	−0.38	0.7035
14	rs17756404	68601208	A	1.27	2.82	0.0048	1.14	1.85	0.0647
14	rs12878761	68601648	A	1.18	1.12	0.2391	1.08	0.66	0.5114

Significant SNPs were highlighted in bold. In this table we showed the results of statistical results when SNPs were coded in additive model.

**Table 3:** Summarized results of association analyses between seven SNPs and four clinical assessments of RA with combined RA patients sample from both discovery and validation stages.

SNP	BP	A1	BETA_DAS28	STAT_DAS28	*P*_DAS28	BETA_VAS	STAT_VAS	*P*_VAS	OR_EA	STAT_EA	*P*_EA	OR_ERO	STAT_ERO	*P*_ERO
rs911263	68286876	C	0.0048	0.11	0.9126	−0.07	−0.28	0.7766	0.92	−0.31	0.7569	**0.52**	**−4.18**	**2.89 × 10**^**−5**^
rs911256	68306863	C	−0.0061	−0.12	0.9054	−0.14	−0.49	0.6213	1.10	0.36	0.7170	0.71	−1.89	0.0594
rs17105837	68562024	A	0.0374	1.39	0.1647	0.25	1.63	0.1046	0.77	−1.70	0.0887	1.05	0.47	0.6378
rs2525504	68562256	G	0.0127	0.47	0.6420	0.10	0.67	0.5053	0.67	−2.48	0.0132	1.00	0.00	0.9963
rs4531674	68600026	A	0.0258	0.63	0.5292	0.13	0.55	0.5850	0.95	−0.21	0.8345	0.89	−0.78	0.4339
rs17756404	68601208	A	0.0188	0.72	0.4703	0.13	0.84	0.3985	0.81	−1.45	0.1484	1.03	0.32	0.7509
rs12878761	68601648	A	0.0406	0.95	0.3414	0.27	1.09	0.2755	0.96	−0.15	0.8784	1.01	0.03	0.9732

The four clinical assessments, VAS, DAS28, extra-articular and erosion were indicated as VAS, DAS28, EA and ERO, respectively. The significant results were indicated in bold.
