# Supplementary material for: Polymorphisms of RAD51B are associated with rheumatoid arthritis and erosion in rheumatoid arthritis patients
Source: Sci Rep. 2017 Mar 31;7:45876. doi: 10.1038/srep45876 (PMC5374468; doi:10.1038/srep45876)
Supplement: Supplementary Information [file srep45876-s1.doc]

***Title***: Polymorphisms of *RAD51B* are associated with rheumatoid arthritis and erosion in rheumatoid arthritis patients

***Author names and affiliations***: Liqiang Zhi ^a*^, Shuxin Yao ^a*^, Wenlong Ma ^b^, Weijie Zhang ^a^, Honggan Chen ^b^,Meng Li ^c^, Jianbing Ma ^a^

^a^ Department of Joint Surgery, Honghui Hospital, Xi’an Jiaotong University Health Science Center, Xi’an, Shaanxi, China;

^b^ Department of Hip Injury and Disease, Orthopedic Hospital of Henan Province, Qiming South Road No.82, Luoyang, Henan, China;

^c^ Department of Orthopedics, the First Affiliated Hospital, Xi'an Jiaotong University, Xi’an, Shaanxi, China.

* These authors contributed equally to this work.

***Corresponding Author***:

Jianbing Ma, M.D. & Ph.D., Department of Joint Surgery, Honghui Hospital, Xi’an Jiaotong University Health Science Center, No.555, Youyi East Road, Xi'an, Shaanxi, China, 710054.

Tel: 86-29-88418009; Fax: 86-29-62818386; E-mail: majianbing2000@163.com

Table S1. The information of the SNPs that were selected for genotyping.

| CHR | SNP | POS | ALLELE | FUNC | GENE | MAF | HWE |
| --- | --- | --- | --- | --- | --- | --- | --- |
| 14 | rs8018709 | 67821166 | C/G | intron | *RAD51B* | 0.089 | 0.31 |
| 14 | rs8007989 | 67849003 | G/T | intron | *RAD51B* | 0.091 | 0.23 |
| 14 | rs1951409 | 67998430 | C/G | intron | *RAD51B* | 0.084 | 1 |
| 14 | rs2208596 | 68033447 | C/T | intron | *RAD51B* | 0.128 | 0.78 |
| 14 | rs7151235 | 68094917 | A/C | intron | *RAD51B* | 0.112 | 0.75 |
| 14 | rs12880237 | 68155101 | A/G | intron,near-gene-3 | *RAD51B* | 0.235 | 1 |
| 14 | rs1536456 | 68208076 | C/T | intron | *RAD51B* | 0.309 | 0.94 |
| 14 | rs1314917 | 68244814 | A/C | intron | *RAD51B* | 0.487 | 0.95 |
| 14 | rs4899234 | 68264492 | A/G | intron | *RAD51B* | 0.267 | 1 |
| 14 | rs6573823 | 68266915 | A/G | intron | *RAD51B* | 0.164 | 1 |
| 14 | rs6573824 | 68272207 | C/G | intron | *RAD51B* | 0.453 | 0.95 |
| 14 | rs12432917 | 68277706 | C/T | intron | *RAD51B* | 0.087 | 0.84 |
| 14 | rs911263 | 68286876 | A/G | intron | *RAD51B* | 0.140 | 0.81 |
| 14 | rs911256 | 68306863 | C/G | intron | *RAD51B* | 0.072 | 0.64 |
| 14 | rs4902566 | 68326837 | C/T | intron | *RAD51B* | 0.476 | 0.95 |
| 14 | rs4902574 | 68370708 | G/T | intron | *RAD51B* | 0.405 | 0.95 |
| 14 | rs7359088 | 68412219 | A/G | intron | *RAD51B* | 0.115 | 1 |
| 14 | rs17105601 | 68417974 | C/T | intron | *RAD51B* | 0.077 | 0.37 |
| 14 | rs2753404 | 68445969 | A/C | intron | *RAD51B* | 0.443 | 0.95 |
| 14 | rs10467820 | 68452299 | A/T | intron | *RAD51B* | 0.474 | 0.95 |
| 14 | rs757369 | 68506829 | A/G | intron | *RAD51B* | 0.438 | 0.84 |
| 14 | rs2078316 | 68507791 | A/G | intron | *RAD51B* | 0.336 | 1 |
| 14 | rs2842346 | 68508657 | A/G | intron | *RAD51B* | 0.114 | 0.75 |
| 14 | rs10135398 | 68518889 | A/G | intron | *RAD51B* | 0.484 | 0.95 |
| 14 | rs7140266 | 68538088 | C/T | intron | *RAD51B* | 0.320 | 1 |
| 14 | rs2842333 | 68541976 | A/G | intron | *RAD51B* | 0.257 | 1 |
| 14 | rs17105837 | 68562024 | A/G | intron | *RAD51B* | 0.387 | 0.95 |
| 14 | rs2525504 | 68562256 | A/G | intron | *RAD51B* | 0.400 | 1 |
| 14 | rs6573841 | 68570804 | C/T | intron | *RAD51B* | 0.111 | 1 |
| 14 | rs7146456 | 68572264 | A/G | intron | *RAD51B* | 0.223 | 0.93 |
| 14 | rs1290997 | 68576496 | G/T | intron | *RAD51B* | 0.373 | 1 |
| 14 | rs2253168 | 68583748 | C/T | intron | *RAD51B* | 0.444 | 1 |
| 14 | rs2253317 | 68585686 | C/G | intron | *RAD51B* | 0.298 | 1 |
| 14 | rs2256206 | 68585707 | A/G | intron | *RAD51B* | 0.314 | 0.82 |
| 14 | rs2256224 | 68586030 | A/G | intron | *RAD51B* | 0.417 | 1 |
| 14 | rs8008333 | 68586480 | A/G | intron | *RAD51B* | 0.298 | 1 |
| 14 | rs2257022 | 68592523 | C/T | intron | *RAD51B* | 0.315 | 1 |
| 14 | rs4531674 | 68600026 | A/G | intron | *RAD51B* | 0.113 | 1 |
| 14 | rs17756404 | 68601208 | A/G | intron | *RAD51B* | 0.409 | 1 |
| 14 | rs12878761 | 68601648 | A/G | intron | *RAD51B* | 0.097 | 0.85 |
| 14 | rs2236185 | 68604047 | C/T | intron | *RAD51B* | 0.395 | 0.95 |
| 14 | rs4262880 | 68605219 | A/G | intron,near-gene-3 | *RAD51B* | 0.165 | 1 |
| 14 | rs4902609 | 68606012 | C/T | intron,ncRNA | *RAD51B* | 0.121 | 1 |
| 14 | rs10137893 | 68606161 | C/T | intron,ncRNA | *RAD51B* | 0.281 | 0.87 |
| 14 | rs10151284 | 68606525 | A/C | intron | *RAD51B* | 0.236 | 1 |
| 14 | rs10143573 | 68607299 | C/T | intron | *RAD51B* | 0.464 | 0.95 |
| 14 | rs17105992 | 68607744 | A/G | intron | *RAD51B* | 0.365 | 0.89 |
| 14 | rs4902611 | 68607814 | A/G | intron | *RAD51B* | 0.406 | 0.84 |
| 14 | rs2331780 | 68618996 | A/G | intron,near-gene-3 | *RAD51B* | 0.129 | 1 |
| 14 | rs17106046 | 68621147 | C/T | intron,untranslated-3 | *RAD51B* | 0.491 | 0.85 |
| 14 | rs10483815 | 68622274 | A/T | intron,untranslated-3 | *RAD51B* | 0.216 | 1 |
| 14 | rs17756627 | 68623720 | A/T | intron | *RAD51B* | 0.224 | 0.93 |
| 14 | rs12893578 | 68637955 | A/G | coding-synon,intron,ncRNA | *RAD51B* | 0.218 | 0.70 |
| 14 | rs12433279 | 68649343 | A/G | intron | *RAD51B* | 0.102 | 1 |
| 14 | rs17835140 | 68652149 | A/G | intron,near-gene-5 | *RAD51B* | 0.134 | 0.89 |
| 14 | rs7350738 | 68661473 | A/T | intron | *RAD51B* | 0.152 | 1 |
| 14 | rs7141573 | 68669036 | A/G | intron,near-gene-5 | *RAD51B* | 0.113 | 1 |
| 14 | rs17106125 | 68670164 | A/C | intron,near-gene-5 | *RAD51B* | 0.100 | 1 |
| 14 | rs8021168 | 68671294 | C/T | intron | *RAD51B* | 0.337 | 1 |
| 14 | rs10143275 | 68674771 | A/G | intron | *RAD51B* | 0.403 | 0.89 |
| 14 | rs1884807 | 68681581 | C/T | intron | *RAD51B* | 0.235 | 1 |
| 14 | rs4899251 | 68683097 | C/T | intron,ncRNA,untranslated-3 | *RAD51B* | 0.303 | 1 |

MAF: minor allele frequency in the discovery sample

HWE: *P* values of Hardy-Weinberg Equilibrium tests in the discovery sample

Table S2. Summarized results of power analysis.

| MAF/OR | 0.05 | 0.1 | 0.15 | 0.2 | 0.25 | 0.3 |
| --- | --- | --- | --- | --- | --- | --- |
| 1.1 | 0.0738 | 0.111 | 0.1138 | 0.0986 | 0.08655 | 0.07708 |
| 1.2 | 0.1157 | 0.3073 | 0.3144 | 0.2481 | 0.1957 | 0.1553 |
| 1.3 | 0.203 | 0.5906 | 0.5953 | 0.473 | 0.3654 | 0.2776 |
| 1.4 | 0.3259 | **0.8268** | **0.825** | 0.6966 | 0.5564 | 0.4253 |
| 1.5 | 0.4718 | **0.9481** | **0.9444** | **0.8559** | 0.7242 | 0.5733 |

Statistical power greater than 0.8 were shown in bold.

Table S3. Single marker based analyses in discovery stage.

| CHR | SNP | BP | A1 | OR_ADD | STAT_ADD | *P*_ADD | OR_DOM | STAT_DOM | *P*_DOM | OR_REC | STAT_REC | *P*_REC |
| --- | --- | --- | --- | --- | --- | --- | --- | --- | --- | --- | --- | --- |
| 14 | rs8018709 | 67821166 | G | 1.08 | 0.51 | 0.6131 | 1.08 | 0.46 | 0.6433 | 1.20 | 0.33 | 0.7392 |
| 14 | rs8007989 | 67849003 | G | 1.08 | 0.53 | 0.5949 | 1.09 | 0.54 | 0.5907 | 1.10 | 0.17 | 0.8636 |
| 14 | rs1951409 | 67998430 | G | 0.96 | -0.24 | 0.8077 | 0.96 | -0.27 | 0.7853 | 1.04 | 0.05 | 0.9581 |
| 14 | rs2208596 | 68033447 | A | 0.95 | -0.37 | 0.7080 | 0.96 | -0.30 | 0.7608 | 0.84 | -0.37 | 0.7130 |
| 14 | rs7151235 | 68094917 | A | 1.03 | 0.22 | 0.8261 | 1.03 | 0.18 | 0.8554 | 1.11 | 0.21 | 0.8350 |
| 14 | rs12880237 | 68155101 | A | 0.97 | -0.34 | 0.7361 | 0.97 | -0.29 | 0.7707 | 0.93 | -0.26 | 0.7981 |
| 14 | rs1536456 | 68208076 | G | 1.04 | 0.38 | 0.7065 | 1.03 | 0.25 | 0.8016 | 1.09 | 0.41 | 0.6816 |
| 14 | rs1314917 | 68244814 | G | 1.02 | 0.28 | 0.7820 | 1.03 | 0.19 | 0.8457 | 1.04 | 0.26 | 0.7964 |
| 14 | rs4899234 | 68264492 | A | 0.97 | -0.37 | 0.7120 | 0.96 | -0.38 | 0.7009 | 0.97 | -0.15 | 0.8782 |
| 14 | rs6573823 | 68266915 | A | 1.04 | 0.38 | 0.7041 | 1.05 | 0.38 | 0.7047 | 1.06 | 0.15 | 0.8781 |
| 14 | rs6573824 | 68272207 | C | 1.03 | 0.35 | 0.7266 | 1.04 | 0.31 | 0.7533 | 1.04 | 0.25 | 0.8001 |
| 14 | rs12432917 | 68277706 | T | 0.96 | -0.29 | 0.7699 | 0.94 | -0.39 | 0.6962 | 1.20 | 0.29 | 0.7681 |
| 14 | rs911263 | 68286876 | C | 0.58 | -4.02 | 5.79E-05 | 0.57 | -3.83 | 1.26E-04 | 0.29 | -2.04 | 0.0411 |
| 14 | rs911256 | 68306863 | C | 0.93 | -0.44 | 0.6635 | 0.91 | -0.53 | 0.5979 | 1.20 | 0.26 | 0.7968 |
| 14 | rs4902566 | 68326837 | C | 0.98 | -0.25 | 0.8045 | 0.97 | -0.21 | 0.8371 | 0.97 | -0.20 | 0.8430 |
| 14 | rs4902574 | 68370708 | G | 0.97 | -0.35 | 0.7297 | 0.96 | -0.32 | 0.7491 | 0.96 | -0.24 | 0.8142 |
| 14 | rs7359088 | 68412219 | G | 1.05 | 0.34 | 0.7361 | 1.04 | 0.27 | 0.7892 | 1.20 | 0.37 | 0.7141 |
| 14 | rs17105601 | 68417974 | T | 0.93 | -0.44 | 0.6598 | 0.93 | -0.44 | 0.6601 | 0.91 | -0.15 | 0.8836 |
| 14 | rs2753404 | 68445969 | G | 1.02 | 0.26 | 0.7972 | 1.03 | 0.24 | 0.8083 | 1.03 | 0.17 | 0.8633 |
| 14 | rs10467820 | 68452299 | A | 0.99 | -0.18 | 0.8597 | 0.98 | -0.14 | 0.8903 | 0.98 | -0.15 | 0.8798 |
| 14 | rs757369 | 68506829 | G | 1.02 | 0.28 | 0.7757 | 1.02 | 0.16 | 0.8696 | 1.05 | 0.31 | 0.7531 |
| 14 | rs2078316 | 68507791 | A | 0.98 | -0.26 | 0.7959 | 0.98 | -0.21 | 0.8356 | 0.96 | -0.22 | 0.8255 |
| 14 | rs2842346 | 68508657 | T | 1.05 | 0.34 | 0.7365 | 1.05 | 0.31 | 0.7564 | 1.11 | 0.21 | 0.8335 |
| 14 | rs10135398 | 68518889 | A | 1.03 | 0.38 | 0.7058 | 1.05 | 0.35 | 0.7275 | 1.04 | 0.27 | 0.7898 |
| 14 | rs7140266 | 68538088 | T | 0.97 | -0.36 | 0.7221 | 0.97 | -0.27 | 0.7875 | 0.94 | -0.33 | 0.7412 |
| 14 | rs2842333 | 68541976 | C | 0.97 | -0.35 | 0.7284 | 0.97 | -0.27 | 0.7890 | 0.92 | -0.33 | 0.7429 |
| 14 | rs17105837 | 68562024 | A | 0.98 | -0.28 | 0.7774 | 0.98 | -0.14 | 0.8922 | 0.94 | -0.36 | 0.7177 |
| 14 | rs2525504 | 68562256 | G | 0.78 | -2.78 | 0.0055 | 0.76 | -2.20 | 0.0278 | 0.66 | -2.36 | 0.0181 |
| 14 | rs6573841 | 68570804 | T | 1.05 | 0.38 | 0.7022 | 1.04 | 0.27 | 0.7881 | 1.32 | 0.54 | 0.5860 |
| 14 | rs7146456 | 68572264 | A | 0.96 | -0.40 | 0.6893 | 0.97 | -0.22 | 0.8225 | 0.85 | -0.58 | 0.5602 |
| 14 | rs1290997 | 68576496 | T | 1.03 | 0.37 | 0.7120 | 1.04 | 0.29 | 0.7736 | 1.06 | 0.32 | 0.7461 |
| 14 | rs2253168 | 68583748 | G | 1.03 | 0.36 | 0.7159 | 1.05 | 0.39 | 0.6961 | 1.03 | 0.19 | 0.8505 |
| 14 | rs2253317 | 68585686 | C | 1.04 | 0.40 | 0.6900 | 1.05 | 0.38 | 0.7022 | 1.05 | 0.24 | 0.8140 |
| 14 | rs2256206 | 68585707 | C | 0.94 | -0.64 | 0.5209 | 0.93 | -0.59 | 0.5583 | 0.92 | -0.44 | 0.6633 |
| 14 | rs2256224 | 68586030 | T | 0.98 | -0.26 | 0.7948 | 0.97 | -0.26 | 0.7960 | 0.98 | -0.16 | 0.8765 |
| 14 | rs8008333 | 68586480 | G | 1.04 | 0.44 | 0.6595 | 1.06 | 0.45 | 0.6510 | 1.04 | 0.21 | 0.8360 |
| 14 | rs2257022 | 68592523 | G | 0.98 | -0.27 | 0.7835 | 0.98 | -0.17 | 0.8627 | 0.94 | -0.32 | 0.7526 |
| 14 | rs4531674 | 68600026 | A | 1.05 | 0.38 | 0.7016 | 1.05 | 0.33 | 0.7437 | 1.18 | 0.33 | 0.7389 |
| 14 | rs17756404 | 68601208 | A | 1.27 | 2.82 | 0.0048 | 1.30 | 2.08 | 0.0374 | 1.48 | 2.60 | 0.0092 |
| 14 | rs12878761 | 68601648 | A | 1.18 | 1.18 | 0.2391 | 1.19 | 1.14 | 0.2538 | 1.37 | 0.50 | 0.6147 |
| 14 | rs2236185 | 68604047 | A | 0.98 | -0.25 | 0.8053 | 0.97 | -0.25 | 0.8052 | 0.98 | -0.14 | 0.8865 |
| 14 | rs4262880 | 68605219 | A | 1.05 | 0.41 | 0.6831 | 1.03 | 0.23 | 0.8198 | 1.26 | 0.67 | 0.5035 |
| 14 | rs4902609 | 68606012 | C | 0.95 | -0.37 | 0.7078 | 0.97 | -0.23 | 0.8204 | 0.68 | -0.67 | 0.5057 |
| 14 | rs10137893 | 68606161 | C | 0.96 | -0.39 | 0.6940 | 0.95 | -0.43 | 0.6680 | 0.97 | -0.14 | 0.8923 |
| 14 | rs10151284 | 68606525 | C | 0.97 | -0.35 | 0.7259 | 0.97 | -0.28 | 0.7806 | 0.92 | -0.32 | 0.7495 |
| 14 | rs10143573 | 68607299 | C | 1.02 | 0.28 | 0.7832 | 1.04 | 0.30 | 0.7638 | 1.02 | 0.14 | 0.8879 |
| 14 | rs17105992 | 68607744 | A | 0.98 | -0.22 | 0.8262 | 0.98 | -0.18 | 0.8557 | 0.97 | -0.18 | 0.8589 |
| 14 | rs4902611 | 68607814 | A | 1.05 | 0.53 | 0.5941 | 1.07 | 0.53 | 0.5965 | 1.05 | 0.32 | 0.7512 |
| 14 | rs2331780 | 68618996 | A | 1.10 | 0.78 | 0.4350 | 1.09 | 0.60 | 0.5480 | 1.45 | 0.87 | 0.3846 |
| 14 | rs17106046 | 68621147 | T | 1.03 | 0.29 | 0.7704 | 1.04 | 0.27 | 0.7900 | 1.03 | 0.21 | 0.8336 |
| 14 | rs10483815 | 68622274 | T | 1.04 | 0.36 | 0.7154 | 1.03 | 0.20 | 0.8417 | 1.16 | 0.54 | 0.5869 |
| 14 | rs17756627 | 68623720 | T | 1.02 | 0.23 | 0.8166 | 1.02 | 0.14 | 0.8857 | 1.09 | 0.30 | 0.7607 |
| 14 | rs12893578 | 68637955 | A | 1.04 | 0.40 | 0.6883 | 1.02 | 0.19 | 0.8516 | 1.19 | 0.66 | 0.5086 |
| 14 | rs12433279 | 68649343 | G | 1.10 | 0.71 | 0.4793 | 1.10 | 0.63 | 0.5305 | 1.36 | 0.54 | 0.5887 |
| 14 | rs17835140 | 68652149 | G | 1.06 | 0.50 | 0.6142 | 1.07 | 0.47 | 0.6382 | 1.14 | 0.30 | 0.7673 |
| 14 | rs7350738 | 68661473 | T | 0.97 | -0.27 | 0.7900 | 0.96 | -0.28 | 0.7770 | 0.98 | -0.05 | 0.9590 |
| 14 | rs7141573 | 68669036 | G | 1.07 | 0.51 | 0.6084 | 1.07 | 0.46 | 0.6475 | 1.21 | 0.37 | 0.7084 |
| 14 | rs17106125 | 68670164 | A | 1.14 | 0.94 | 0.3486 | 1.14 | 0.88 | 0.3781 | 1.34 | 0.52 | 0.6022 |
| 14 | rs8021168 | 68671294 | C | 0.97 | -0.33 | 0.7412 | 0.97 | -0.27 | 0.7843 | 0.95 | -0.27 | 0.7891 |
| 14 | rs10143275 | 68674771 | A | 1.03 | 0.34 | 0.7313 | 1.03 | 0.23 | 0.8171 | 1.06 | 0.35 | 0.7291 |
| 14 | rs1884807 | 68681581 | A | 0.97 | -0.35 | 0.7253 | 0.96 | -0.32 | 0.7494 | 0.94 | -0.23 | 0.8162 |
| 14 | rs4899251 | 68683097 | C | 0.96 | -0.39 | 0.6958 | 0.96 | -0.31 | 0.7533 | 0.93 | -0.34 | 0.7371 |

Additive, dominant and recessive model for SNP coding were indicated as ADD, DOM and REC, respectively.

Table S4. Haplotype analyses of two LD blocks using data from discovery stage.

| LOCUS | HAPLOTYPE | F_A | F_U | CHISQ | DF | P | SNPS |
| --- | --- | --- | --- | --- | --- | --- | --- |
| H1 | OMNIBUS | NA | NA | 0.90 | 2 | 0.6368 | rs1290997|rs2253168 |
| H1 | TG | 0.37 | 0.37 | 0.01 | 1 | 0.9297 | rs1290997|rs2253168 |
| H1 | GG | 0.09 | 0.08 | 0.81 | 1 | 0.3670 | rs1290997|rs2253168 |
| H1 | GA | 0.54 | 0.56 | 0.33 | 1 | 0.5638 | rs1290997|rs2253168 |
| H2 | OMNIBUS | NA | NA | 5.98 | 2 | 0.0503 | rs17105992|rs4902611 |
| H2 | AA | 0.35 | 0.36 | 0.18 | 1 | 0.6744 | rs17105992|rs4902611 |
| H2 | GA | 0.07 | 0.04 | 5.98 | 1 | 0.0145 | rs17105992|rs4902611 |
| H2 | GG | 0.58 | 0.59 | 0.45 | 1 | 0.5030 | rs17105992|rs4902611 |

Table S5. Haplotype analyses based on combined data for all 7 SNPs selected for genotyping in validation stage.

| LOCUS | HAPLOTYPE | F_A | F_U | CHISQ | DF | P | SNPS |
| --- | --- | --- | --- | --- | --- | --- | --- |
| H1 | OMNIBUS | NA | NA | 40.31 | 2 | 1.76×10^-9^ | rs911263|rs911256 |
| H1 | CC | 0.06 | 0.07 | 2.71 | 1 | 0.10 | rs911263|rs911256 |
| H1 | CG | 0.04 | 0.08 | 35.90 | 1 | 2.08×10^-9^ | rs911263|rs911256 |
| H1 | TG | 0.90 | 0.85 | 32.17 | 1 | 1.41×10^-8^ | rs911263|rs911256 |
| H2 | OMNIBUS | NA | NA | 32.12 | 7 | 3.86×10^-5^ | rs17105837|rs2525504|rs4531674|rs17756404|rs12878761 |
| H2 | AGAAA | 0.03 | 0.03 | 0.17 | 1 | 0.68 | rs17105837|rs2525504|rs4531674|rs17756404|rs12878761 |
| H2 | GAAAA | 0.05 | 0.05 | 0.47 | 1 | 0.49 | rs17105837|rs2525504|rs4531674|rs17756404|rs12878761 |
| H2 | GAAAG | 0.02 | 0.02 | 1.67 | 1 | 0.20 | rs17105837|rs2525504|rs4531674|rs17756404|rs12878761 |
| H2 | AGGAG | 0.12 | 0.11 | 1.31 | 1 | 0.25 | rs17105837|rs2525504|rs4531674|rs17756404|rs12878761 |
| H2 | GAGAG | 0.21 | 0.17 | 17.19 | 1 | 3.39×10^-5^ | rs17105837|rs2525504|rs4531674|rs17756404|rs12878761 |
| H2 | AGGGG | 0.22 | 0.24 | 5.38 | 1 | 0.02 | rs17105837|rs2525504|rs4531674|rs17756404|rs12878761 |
| H2 | GGGGG | 0.01 | 0.02 | 9.87 | 1 | 0.00 | rs17105837|rs2525504|rs4531674|rs17756404|rs12878761 |
| H2 | GAGGG | 0.34 | 0.35 | 1.33 | 1 | 0.25 | rs17105837|rs2525504|rs4531674|rs17756404|rs12878761 |
